# The World of Antiherpetic Vaccines and Drugs, 2022

**DOI:** 10.3390/v14050850

**Published:** 2022-04-20

**Authors:** Barry J. Margulies

**Affiliations:** Towson University Herpes Virus Lab, Department of Biological Sciences, Towson University, Towson, MD 21252, USA; bjmarg@alum.mit.edu; Tel.: +1-(410)-704-5019; Fax: +1-(410)-704-2405

The world of antiherpetics has grown by leaps and bounds since the discovery of what would become the first antiherpetic drug in 1964 [1]. We now have licensed vaccines to prevent veterinary diseases associated with equine herpesvirus-1 [2,3], pseudorabies virus [4,5], bovine herpesvirus-1 [6], feline herpesvirus-1 [7], infectious laryngotracheitis virus/gallid herpesvirus-1 [8], Marek’s Disease Virus [9,10], and cyprinid herpesvirus-3 [8], all herpesviruses that cause significant morbidity and mortality. We also have excellent vaccines, Varivax/Zostavax [11,12] and Shingrix [13], to prevent chicken pox and shingles in humans. There are many licensed drugs, e.g., letermovir [14] and acyclovir [15,16], to prevent herpesvirus outbreaks. However, we are constantly discovering new ways to attack herpesviruses, including novel methods aiming at prophylactic and therapeutic approaches.

In this Special Issue, we begin with the mundane, the currently applicable anti-herpes simplex drug market [17,18]; these articles cover the pharmaceutical armaments available today to deal with recurrent HSV outbreaks. Current treatments for Kaposi’s sarcoma are addressed, especially drugs that combat the cancer itself [19]. We also present information on the search for novel antiherpetics [20,21,22] to combat infection with other human herpesviruses. However, none of the chemotherapeutic interventions discussed deal with preventing a herpes infection.

Novel work in herpesvirus vaccines is extending the original work of anti-VZV work. As was seen with current anti-SARS-CoV-2 approaches, vaccines to combat HSV-2 based on recombinant protein subunits or mRNAs are discussed [23]. This exciting research may be a major step forward in preventing primary infection. Similarly, live, attenuated herpesviruses can be used to elicit durable, lasting, immune responses to diminish the number and severity of reactivations [24,25]. A fourth paper in this issue [26] addresses limitations that may be encountered when working with live, attenuated herpesviruses.

While the established pharmacopeia typically uses synthetic molecules that have been discovered through direct design or high-throughout screens, a number of researchers have taken the path of exploring natural compounds. This Special Issue describes multiple natural products used to prevent primary infection in vitro and in vivo [27]. Furthermore, and of greater consequence, they demonstrate the incredible safety of these compounds in animal models, an important step towards testing these compounds in Phase I safety trials.

It is with great pleasure that we provide this Special Issue, presenting both current and forward-looking concepts in antiviral intervention, and we welcome your feedback.
